# History of an Interesting Case of Strangulated Hernia

**Published:** 1820-12

**Authors:** Edw. Th. Bond

**Affiliations:** Member of the Royal College of Surgeons.


FOR THE LONDON MEDICAL AND PHYSICAL JOURNAL.

### *History of an interesting Case of Strangulated Hernia.*

By Mr. EDW. TH. BOND, Member of the Royal College of Surgeons.

IN most cases of hernia there is, perhaps, little unusual, either to excite peculiar attention while under treatment, or to elicit a communication of them afterwards: each one is generally, in its leading features, little more than a "languid, leaden, iteration" of others which have preceded it; and the accounts of them are histories which we feel often inclined to lay down in weariness, and, with the words of Æneas upon our lips, the "iterumque iterumque," if not of disappointment, of satiety. The following one is open probably to objections

---

of this nature; but the giant strides with which the severer and more fatal symptoms made their appearance gave it, to me, an interest which it certainly would not otherwise have possessed; and though, to older and more experienced practitioners, who have, it is to be presumed, seen many such, it may appear like "the uses of this world" to Hamlet, "flat and unprofitable," there are others young, like myself, in the profession, and the same circumstances which raised an interest for the case in me, may possibly give to its recital something of novelty for them. Its history is given from the notes entered in my case-book at the time.

George Taylor, a ribbon-weaver, aged thirty-six, who had not long recovered from an attack of jaundice, a man of spare habit, thin and pale, had been for some time subject to rupture, but experienced of late no inconvenience from it until yesterday morning, (Sunday, Sept. 24,) about nine o'clock, when it came down directly after stool, and he was immediately seized with very severe pain, both in the consequent tumor and in the abdomen, which continued unremittingly through the day; much tension of the parts and sickness gradually supervening. At night he had six or seven grains of calomel, with a dose of sulphate of magnesia, as his complaints were described to be pain in the bowels with costiveness; and I saw him for the first time about nine this morning, (Monday 25th,) in consequence of a message received between seven and eight o'clock; and now it was that I was first told of the existence of hernia. His pulse was small and scarcely perceptible; his countenance pallid, and indicative of great distress; his tongue dry, and his eyes sunken; but this last was perhaps in some degree his natural feature. His sickness was constant and distressing, but there was no hiccup; tension of the abdomen great; yet, though he complained of incessant pain, pressure made either on the abdomen or tumor itself did not seem so difficult to be borne as might *a priori* have been expected.

Symptoms such as these, gave, of course, great reason to fear that gangrene had already commenced; but, as the time since strangulation first took place was so very short, scarcely twenty-four hours, I was not deterred from giving my patient the chance of the operation, which, as it was evident that it was the only hope to be rationally confided in, I lost no time in having recourse to: it was performed by, at the farthest, eleven o'clock. Other measures (save, indeed, the taxis for a few minutes,) I tried none. There was every thing to point out the danger of delay: half an hour now was of consequence; and, in the prosecution of the usual means there was a certainty of their occupying time which, under existing circumstances, was invaluable, with no certainty of their attaining the proposed end,—the relief of the intestine from confinement.

There was not, I think, half an ounce of serum in the herniary sac, which contained, however, a very considerable quantity of much-distended intestine, (from six to seven or eight inches of jejunum or ileum, be it which it might,) with a portion of mesentery; and a considerable part of the gut, about half, was nearly black. After opening the peritoneal sac through its whole extent, I divided the tendon of the external oblique, where it forms the upper margin of the external ring, directly upwards and very freely, but was still unable to return the protruded intestine; and, on making closer examination, I found that there was a second stricture almost immediately (supposing the man in an erect position) behind the first, formed by the upper margin of the internal ring, which, though thus brought down by the descent of the gut so as to make, in fact, nearly one direct opening into the abdominal cavity, formed a separate and distinct stricture. I divided it in the same direction with the former incision, (i. e. directly upwards,) and then returned the intestine, its ascent being accompanied with much gurgling noise. The wound was now dressed in the usual manner, and the man put to bed.

Symptoms did not seem much improved, except that he appeared somewhat more cheerful, and his eye something more lively; but his sickness continued; his pulse, if it was at all, was scarcely to be felt; and he complained of great load in his belly.

I left him, with directions that he should have a purgative enema every hour, and aperient pills by the mouth every two hours, if his stomach would retain them; intending to see him again towards five in the afternoon: but at four he died.

It is not, at any rate, often that a hernia left so completely to itself as this was, hurries over its fatal course with similar rapidity. The whole time from the first formation of stricture until the man's death, did not embrace a period of more than thirty-one hours; until the intestine was freed from confinement by the operation, of not more than twenty-six hours; and gangrene had doubtless, judging from the symptoms combined with the appearance of the gut, commenced some hours previously even to this; probably in twenty hours from the first occurrence of strangulation. The gut was not at all disturbed by vain attempts to reduce it; for aware, on my first seeing him, of the necessity there was for prompt measures, and that only decisive practice could possibly save him, I did not employ the taxis for more than a few minutes, and with the utmost gentleness; losing no time, therefore, in any futile endeavour of the kind, but had recourse to the operation at once, and without the least unnecessary delay: so that the rapidity with which the fatal symptoms came on was not here, as is perhaps sometimes the case, at all accelerated by any long-protracted handling of the tumor, which, let it be managed as judiciously as it may, if it is not effectual in procuring relief, can scarcely fail of increasing the mischief. But the inflammation, produced as it was, and kept up, solely by the severity and closeness of the stricture, acting, it is probable, on a bad habit of body, ran its own rapid career, from its earliest origin to its termination in gangrene, in less than a day; adding thus one case to the many on record, which show how necessary it is that *symptoms*, not the time which may have elapsed since the commencement of the disease, should furnish our rule of practice.

*Nun-Eaton; October 7th,* 1820.

